# Disease Burden and Pattern of Healthcare Utilization Among Pilgrims During Hajj 2024: A Cross‑Sectional Analysis

**DOI:** 10.5334/aogh.4956

**Published:** 2026-03-09

**Authors:** Khulud K. Alharbi, Mashael S. Alfaifi, Ali M. Alzahrani, Ahmad Salah Alkathiri, Tassnym H. Sinky

**Affiliations:** 1 Department of Health Administration and Hospitals, College of Public Health and Health Informatics, Umm Al‑Qura University, Makkah, Saudi Arabia; 2 Department of Epidemiology & Medical Statistic, College of Public Health and Health Informatics, Umm Al‑Qura University, Makkah, Saudi Arabia; 3 Department of Health Promotion and Education, College of Public Health and Health Informatics, Umm Al‑Qura University, Makkah, Saudi Arabia

**Keywords:** Hajj, pilgrims, utilization, pattern, diseases burden

## Abstract

*Background:* The Hajj pilgrimage is one of the largest annual mass gatherings in the world, and it presents unique healthcare issues due to the vast number and diversity of participants. Optimizing the delivery and planning of health services requires an understanding of prevalent diseases and healthcare usage patterns. The aim of the study was to examine the patterns of disease burden and healthcare utilization among 2024 Hajj pilgrims.

*Methods:* This study used a retrospective, descriptive cross‑sectional design. Data from 37,758 adult patient records in the outpatient clinics (OPCs) of the primary healthcare centers (PHCs) and hospitals located at the holy sites (Mena, Arafat, and Muzdalifah) during Hajj 2024 were analyzed. Data covered demographics, nationality, diagnoses, discharge outcomes, and healthcare utilization in holy sites. Patterns and associations were assessed using descriptive statistics and chi‑square testing (*p* < 0.05).

*Results:* Most pilgrims were men (65.5%), and older than 60 years of age (26.4%). They came from more than 100 different countries. The most frequent diagnosis (44.6%) was upper respiratory tract infections (URTIs), which was followed by dermatitis (6.3%), gastrointestinal disorders (7.4%), headaches (7.8%), and musculoskeletal problems (7.3%). Geographically, disease prevalence varied: URTIs were most common in Mena (46.3%), dermatitis peaked in Muzdalifah (14.8%), and heat exhaustion was most common in Arafat (9.4%). Primary care use peaked in Mena (14,500 visits), mirroring pilgrim mobility.

*Conclusion:* The results emphasize the necessity for flexible, data‑driven resource allocation by highlighting the dynamic and site‑specific character of healthcare demands during the Hajj. To improve health outcomes in upcoming Hajj seasons, it is imperative to enhance infection control, heat illness prevention, and culturally competent care, in addition to tailored interventions for older pilgrims and those with chronic illnesses.

## 1. Introduction

The annual Hajj pilgrimage to Mecca is one of the largest mass gatherings in the world, attracting approximately **2–3 million Muslims from more than 180 countries, representing diverse geographic and demographic backgrounds** [1]. This pilgrimage occurs over a fixed period of the Islamic lunar calendar (8–13 Dhul Hijjah), during which pilgrims perform synchronized rituals requiring movement between multiple geographically distinct sites, including Masjid al‑Haram in Makkah, Mina, Arafat, and Muzdalifah. These locations vary in distance, crowd density, and environmental exposure, necessitating rapid population mobilization within confined spaces. The event poses substantial public health challenges and places considerable strain on Saudi Arabia’s healthcare system, making effective and timely planning essential. Managing the health and safety of pilgrims is a critical concern for Saudi health authorities, particularly due to potential risks such as infectious disease outbreaks, heat‑related illnesses, and chronic disease exacerbations [2]. Historically, the Hajj has been recognized as a landmark mass‑gathering event associated with an increased risk of meningococcal disease transmission, with international outbreaks directly leading to the implementation of mandatory quadrivalent meningococcal vaccination policies for pilgrims [3]. In response, Saudi authorities implement a coordinated, multisectoral strategy to ensure comprehensive public health preparedness and management during Hajj [4].

Healthcare services during the Hajj pilgrimage are a critical component of the overall management of this massive event. To accommodate the health needs of millions of pilgrims, the Saudi Ministry of Health establishes an extensive network of healthcare facilities, including hospitals, specialized clinics, and many primary healthcare centers (PHCs) strategically located in key pilgrimage sites. In Al‑Mashaer, one of the central areas for Hajj rituals, there are approximately 75 PHCs that provide essential medical services such as treatment of common illnesses, management of chronic diseases, and health education. PHCs handle most healthcare needs during Hajj, making their efficient operation essential for easing the pressure on higher‑level facilities and ensuring prompt medical services. Healthcare delivery during Hajj follows a structured, tiered model designed to optimize patient flow and resource utilization in a high‑density mass‑gathering environment [5]. Pilgrims may present directly to PHCs for assessment and management of mild‑to‑moderate complaints, or they may present directly to hospital emergency departments for urgent conditions. At the hospital level, patients are triaged upon arrival according to standardized emergency protocols. If the condition is assessed as mild or moderate and does not require immediate emergency intervention or admission, the patient is redirected to hospital outpatient clinics (OPCs) for further evaluation and management [6]. Analyzing their utilization helps inform better planning and resource allocation for healthcare delivery throughout the pilgrimage [7].

Yezli et al. [7] analyzed data from 51 PHCs and found that most pilgrim visits occurred in Mena toward the end of Hajj, with respiratory illnesses being the most frequent diagnosis. They emphasized that such data support evidence‑based planning and resource allocation to improve service delivery during the event. Similarly, Kolivand et al. [8] analyzed Iranian Hajj pilgrims from 2013 to 2022, finding peak mortality in 2015 (509 deaths, mainly men aged 45–70) and highest hospitalizations in 2019 (89,492 cases), with marked provincial differences—insights that can guide targeted, demographic‑ and location‑specific healthcare planning. Complementing these findings, Mirza et al. [9] assessed emergency department usage during Hajj and noted peaks in non‑urgent cases during the day and higher admissions at night, recommending extended primary care hours and better coordination to enhance patient flow and reduce pressure on hospitals. Together, these studies underscore the critical role of utilization data in improving healthcare quality, resource allocation, and operational responsiveness during Hajj.

The Ministry of Health maintains a comprehensive Hajj Health Services database containing demographic, clinical, and outcome data for all registered pilgrims. Despite this valuable resource, few studies have analyzed health services or outcomes at a large scale. Most existing research focuses on limited subgroups, leaving a gap in understanding broader patterns. Utilizing the full national dataset offers an important opportunity to assess the key health challenges of Hajj and help safeguard a more fulfilling and healthier pilgrimage experience for all participants.

This study utilized a retrospective, descriptive cross‑sectional design to analyze data from the Ministry of Health’s Hajj Health Services database, focusing on pilgrims who participated in Hajj during 2024. By examining healthcare utilization patterns across demographic factors, nationality distributions, disease profiles, discharge outcomes, and geographic locations, the research seeks to enhance understanding of the health risks associated with Hajj and support the development of effective health policies and service improvements. Understanding how, when, and where pilgrims access healthcare is crucial for identifying service gaps, peak demand periods, and common medical needs. These insights can guide better planning and allocation of healthcare resources, improve patient flow management, and enable timely decision‑making during critical periods. In this context, the integration of centralized digital dashboards enabling real‑time review of patient data across PHCs and OPCs represents a future‑oriented approach with the potential to strengthen surveillance, coordination, and operational responsiveness during Hajj. Ultimately, the findings will contribute to optimizing healthcare operations, improving the quality of care, and ensuring a safer, more responsive, and fulfilling Hajj experience for all participants.

## 2. Methods

### 2.1 Study design and setting

A retrospective, descriptive cross‑sectional study was conducted in the OPCs of the PHCs and hospitals located at the holy sites (Mena, Arafat, and Muzdalifah) during the 2024 Hajj. There were 46 PHCs and 4 hospitals in Mena, 25 PHCs and 4 hospitals in Arafat, and 4 PHCs in Muzdalifah. All the OPCs operate 24 hours, and pilgrims can access care free of charge by presenting the official Hajj bracelet at healthcare facilities in the holy sites.

### 2.2 Data collection

The Department of Population Health Management (PHM) at Makkah Health Cluster systematically collected data on pilgrims who visited the OPCs located at the holy sites from June 13th to June 17th, 2024. The collected data included patients’ demographic characteristics (age, gender, and nationality), visit date, final diagnosis, diagnosis code, discharge status, and the healthcare facility’s name. This study included patients aged 16 years and older who visited the OPCs at the holy sites and had completed records.

### 2.3 Statistical analysis

Descriptive statistics (e.g., frequency distribution and percentages) were employed to summarize the data and describe patients and common diagnoses encountered at the OPCs at the holy sites during the Hajj of 2024. The Chi‑square test of association was performed to compare patients’ groups based on the outcome of interest (most common diagnoses) and their demographic characteristics (e.g., age, gender, and nationality). Stata/BE 17.0 (Stata Corp, College Station, TX) was used to prepare the data and perform statistical analyses, and Microsoft Power BI was used to create graphs and build visualizations. The significance level was set at *p* < 0.05.

### 2.4 Ethical considerations

Prior to obtaining data from the Makkah Health Cluster, the study was approved from the Institutional Review Board at Saudi Ministry of Health (IRB log No: 24‑100 E).

## 3. Results

A total of 66,693 patient records were initially retrieved for analysis. Following the application of predefined exclusion criteria—namely, restricting the dataset to Hajj pilgrims and excluding records with unspecified gender, age below 16 years, missing data, or unclear nationality or diagnosis—a final sample of 37,758 records was retained for analysis. The results are presented across several key domains, including demographic characteristics, nationality distribution, disease patterns, discharge outcomes, and the geographic distribution of clinical encounters.

### 3.1 Demographic profile of participants

Table 1 presents the demographic characteristics of the study sample, comprising a total of 37,758 participants. The sample was predominantly male, accounting for 65.53% (24,744), while females represented 34.47% (13,014). Most participants were aged above 60 years (26.41%), followed by the 56–60 years (12.32%), 51–55 years (12.12%), and 46–50 years (11.61%) age groups, indicating an older population distribution. Participants aged between 16 and 25 years were the smallest group at only 3.61%. This finding suggests that older pilgrims had a higher utilization of healthcare services, which is likely attributable to the increased prevalence of age‑related comorbidities and greater vulnerability to health complications.

**Table 1:** Demographic characteristics of the study sample and nationalities.

TOTAL NUMBER	37,758	
VARIABLES	N	(%)
**Gender**		
** Male**	24,744	65.53
** Female**	13,014	34.47
**Age (Years)**		
** 16–25**	1,362	3.61
** 26–30**	1,711	4.53
** 31–35**	2,879	7.62
** 36–40**	3,864	10.23
** 41–45**	4,358	11.54
** 46–50**	4,383	11.61
** 51–55**	4,577	12.12
** 56–60**	4,653	12.32
** >60**	9,971	26.41
**Nationality**		
** Egypt**	5,180	13.72
** Pakistan**	4,203	11.13
** India**	3,958	10.48
** Iran**	3,891	10.31
** Saudi Arabia**	3,559	9.43
** Nigeria**	2,756	7.30
** Morocco**	2,230	5.91
** Algeria**	1,459	3.86
** Bangladesh**	1,071	2.84
** Jordan**	851	2.25
** Afghanistan**	832	2.20
** Libya**	812	2.15
** Iraq**	788	2.09
** Yemen**	611	1.62
** Niger**	584	1.55
** Sudan**	494	1.31
** Turkey**	379	1.00
** 104 countries < 1%**	4,100	10.86

### 3.2 Distribution of participants by nationality

The study sample included participants from a wide range of nationalities, as presented in Table 1, reflecting considerable ethnic diversity. The most represented nationality was Egyptian, accounting for 13.72% of the sample, followed by participants from Pakistan (11.13%), India (10.48%), and Iran (10.31%). Saudi nationals comprised 9.43% of the total, while other notable representations included individuals from Nigeria (7.30%), Morocco (5.91%), and Algeria (3.86%). Several other countries, such as Bangladesh, Jordan, Afghanistan, Libya, Iraq, Yemen, Niger, Sudan, and Turkey, each contributed between 1% and 2.84% of the sample. Additionally, 104 nationalities were represented at less than 1% individually, collectively making up 10.86% of the population. This broad representation highlights the multicultural nature of the study population.

### 3.3 Disease distribution among the study population

Table 2 presents the most common diagnoses among patients who attended OPCs across the three holy sites—Mena, Arafat, and Muzdalifah—during the 2024 Hajj season. Of the 37,758 patients analyzed, the most frequently reported condition was **upper respiratory tract infection (URTI)**, accounting for 44.56% of all diagnoses, with the highest proportion observed in Mena (46.33%) and the lowest in Muzdalifah (22.89%). **Headache** was the second most prevalent diagnosis (7.82%), particularly common in Arafat (11.09%) and Muzdalifah (12.95%). **Gastrointestinal conditions**, including colitis, represented 7.35% of diagnoses overall, with a relatively higher incidence in Arafat (10.37%) and Muzdalifah (11.56%). **Musculoskeletal disorders** were also notable, comprising 7.28% of diagnoses, with the highest frequency in Mena (7.75%).

**Table 2:** The most common diagnoses among patients attending the outpatient clinics in the holy sites during the 2024 Hajj.

DIAGNOSIS	ALL (N = 37,758) N (%)	MENA (N = 31,214) N (%)	ARAFAT (N = 5,679) N (%)	MUZDALIFAH (N = 865) N (%)
**Upper respiratory tract infection**	16,824 (44.56)	14,463 (46.33)	2,163 (38.09)	198 (22.89)
**Headache**	2,951 (7.82)	2,209 (7.08)	630 (11.09)	112 (12.95)
**Gastrointestinal and colitis**	2,774 (7.35)	2,085 (6.68)	589 (10.37)	100 (11.56)
**Diseases of the musculoskeletal system**	2,748 (7.28)	2,419 (7.75)	266 (4.68)	63 (7.28)
**Dermatitis**	2,377 (6.30)	2,036 (6.52)	213 (3.75)	128 (14.80)
**Heat exhaustion**	1,371 (3.63)	786 (2.52)	534 (9.40)	51 (5.90)
**Diabetes mellitus**	1,316 (3.49)	1,065 (3.41)	221 (3.89)	30 (3.47)
**Lower back pain**	1,276 (3.38)	1,105 (3.54)	138 (2.43)	33 (3.82)
**Hypertension**	1,037 (2.75)	835 (2.68)	180 (3.17)	22 (2.54)
**Burn of unspecified body region**	806 (2.13)	704 (2.26)	74 (1.30)	28 (3.24)
**Injuries**	787 (2.08)	656 (2.10)	95 (1.67)	36 (4.16)
**Conjunctivitis**	766 (2.03)	655 (2.10)	98 (1.73)	13 (1.50)
**General counseling and advice**	748 (1.98)	554 (1.77)	191 (3.36)	3 (0.35)
**Lower respiratory tract infection**	629 (1.67)	533 (1.71)	84 (1.48)	12 (1.39)
**Bronchial asthma**	606 (1.60)	460 (1.47)	118 (2.08)	28 (3.24)
**Cellulitis**	385 (1.02)	353 (1.13)	30 (0.53)	2 (0.23)
**Disorder of urinary system**	357 (0.95)	296 (0.95)	55 (0.97)	6 (0.69)

Other frequently encountered conditions included **dermatitis** (6.30%), especially prominent in Muzdalifah (14.80%), **heat exhaustion** (3.63%), which peaked in Arafat (9.40%), and **diabetes mellitus** (3.49%), seen consistently across all sites. Additional diagnoses such as **lower back pain** (3.38%), **hypertension** (2.75%), **burns** (2.13%), and **injuries** (2.08%) were also reported, along with **conjunctivitis**, **bronchial asthma**, **lower respiratory tract infections**, **general counseling**, **cellulitis**, and **urinary system disorders**, each constituting less than 2% of the total. The variation in disease prevalence across sites reflects the differing environmental exposures, physical demands, and crowding conditions encountered by pilgrims during various phases of the Hajj.

### 3.4 Patient characteristics

During the 2024 Hajj, URTIs were the most common diagnosis among patients attending OPCs, accounting for 45.98% of males and 41.85% of females, with a significant difference between genders (*p* < 0.001). Gastrointestinal and colitis disorders were more prevalent in females (9.78%) than males (6.07%) (*p* < 0.001), as were musculoskeletal diseases (6.88% among males vs. 8.04% among females, *p* < 0.001). Dermatitis showed a marked male predominance (7.63% vs. 3.75%, *p* < 0.001), whereas heat exhaustion was significantly higher among females (5.33% vs. 2.74%, *p* < 0.001).

Age‑stratified analysis revealed that URTI remained predominant across all age groups, particularly among younger pilgrims (26–30 years: 47.05%), with its prevalence declining in those aged >60 years (41.04%, *p* < 0.001). Gastrointestinal and colitis disorders were most frequent in the 26–30 age group (9.82%, *p* < 0.001), while hypertension and diabetes were markedly higher among older pilgrims (>60 years: HTN 4.33%, DM 5.82%, both *p* < 0.001).

Nationality‑based differences were also observed. URTI rates were highest among pilgrims from Iran (63.20%) and Nigeria (57.84%), while gastrointestinal disorders were most prevalent among Turkish pilgrims (20.05%, *p* < 0.001). Dermatitis was more common among Algerian (10.90%) and Yemeni pilgrims (11.46%), whereas heat exhaustion was most reported among Egyptian (6.49%) and Yemeni pilgrims (11.46%). Diabetes was particularly common among pilgrims from Sudan (8.50%), while hypertension was highest in Iraq (7.11%) (Supplementary Table 1).

### 3.5 Primary healthcare utilization patterns across the Hajj sites and dates

**Figure 1:**
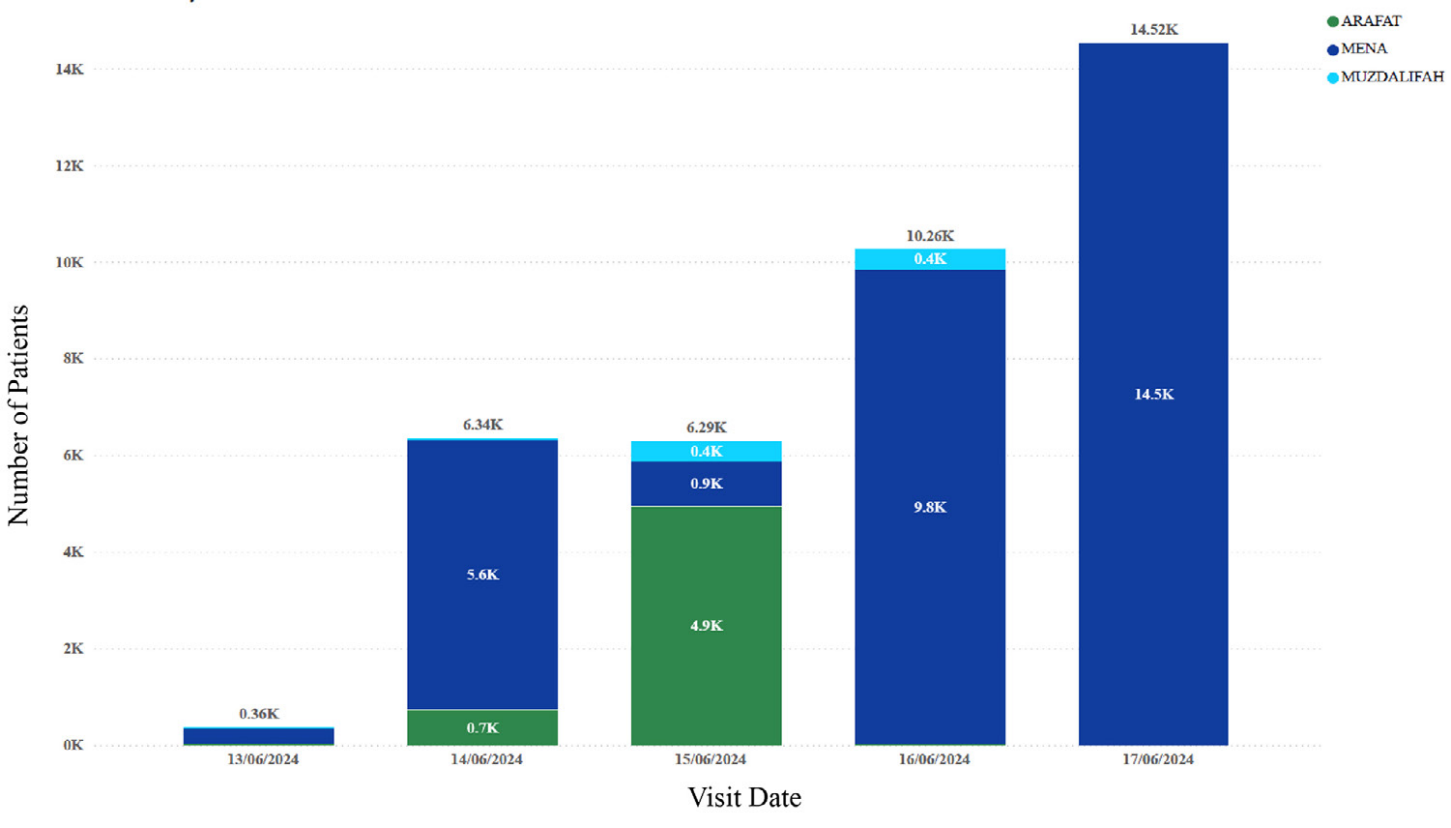
Number of patients visiting the outpatient clinics in the holy sites during the 2024 Hajj.

Figure 1 **illustrates** the utilization patterns of primary healthcare services during the Hajj period from June 13 to June 17, 2024, showing clear correlations with the movement of pilgrims across the holy sites. On June 14 (8th of Dhul Qadah), when most pilgrims were located in Mena, PHCs utilization reached approximately 6.3 thousand visits (5.6K in Mena and 0.7K in Arafat). On June 15, as pilgrims proceeded to Arafat, service utilization shifted accordingly, with 4.9 thousand visits recorded in this location. Following Arafat, many pilgrims moved to Muzdalifah, where the demand for PHC services was comparatively lower. Subsequently, as pilgrims returned to Mena for the remainder of the Hajj, service utilization rose sharply, peaking at 14.5 thousand visits on June 17.

Overall, most patients were managed in **Mena**, followed by **Arafat** and **Muzdalifah**, reflecting the distribution of pilgrims across these sites during the Hajj period. Distinct geographical variations in disease patterns were also evident. For instance, **heat exhaustion** was most frequently reported in Arafat, likely attributable to prolonged outdoor exposure and high ambient temperatures during the pilgrimage rituals performed there. In contrast, **headache**, **gastrointestinal disorders**, and **dermatitis** showed relatively higher proportions in Muzdalifah, which may be related to overnight stays, environmental conditions, and limited access to amenities. **URTIs** were predominantly recorded in Mena, suggesting that crowd density and extended periods spent in shared accommodation facilities may have contributed to increased transmission.

### 3.6 Distribution of major diagnoses among Hajj pilgrims across the Hajj days and locations

**Figure 2:**
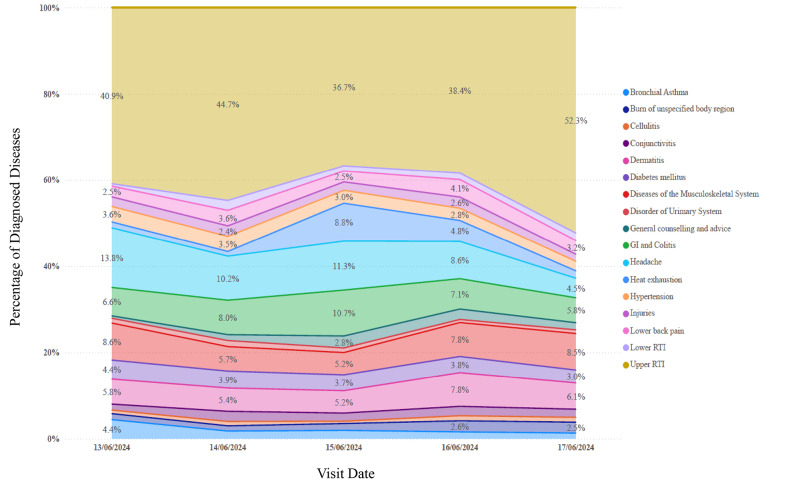
Percentage of diseases diagnosed at the outpatient clinics by visit date during the 2024 Hajj.

The prevalence of diseases varied across the Hajj days, with several conditions showing notable fluctuations in their distribution. **URTIs** consistently accounted for the highest proportion of cases throughout the observed period, peaking at **52.3% on June 17**. On **June 15 (Day of Arafat), heat exhaustion** (8.8%), **headache** (11.3%), and **gastrointestinal disorders (GI)** (10.7%) also showed significant increases, reflecting the environmental and physical demands of that day. **Dermatitis** reached its highest proportion on **June 16 (the day after Arafat)**, accounting for 7.8% of cases, which may be associated with cumulative exposure and exertion (Figure 2).

**Figure 3:**
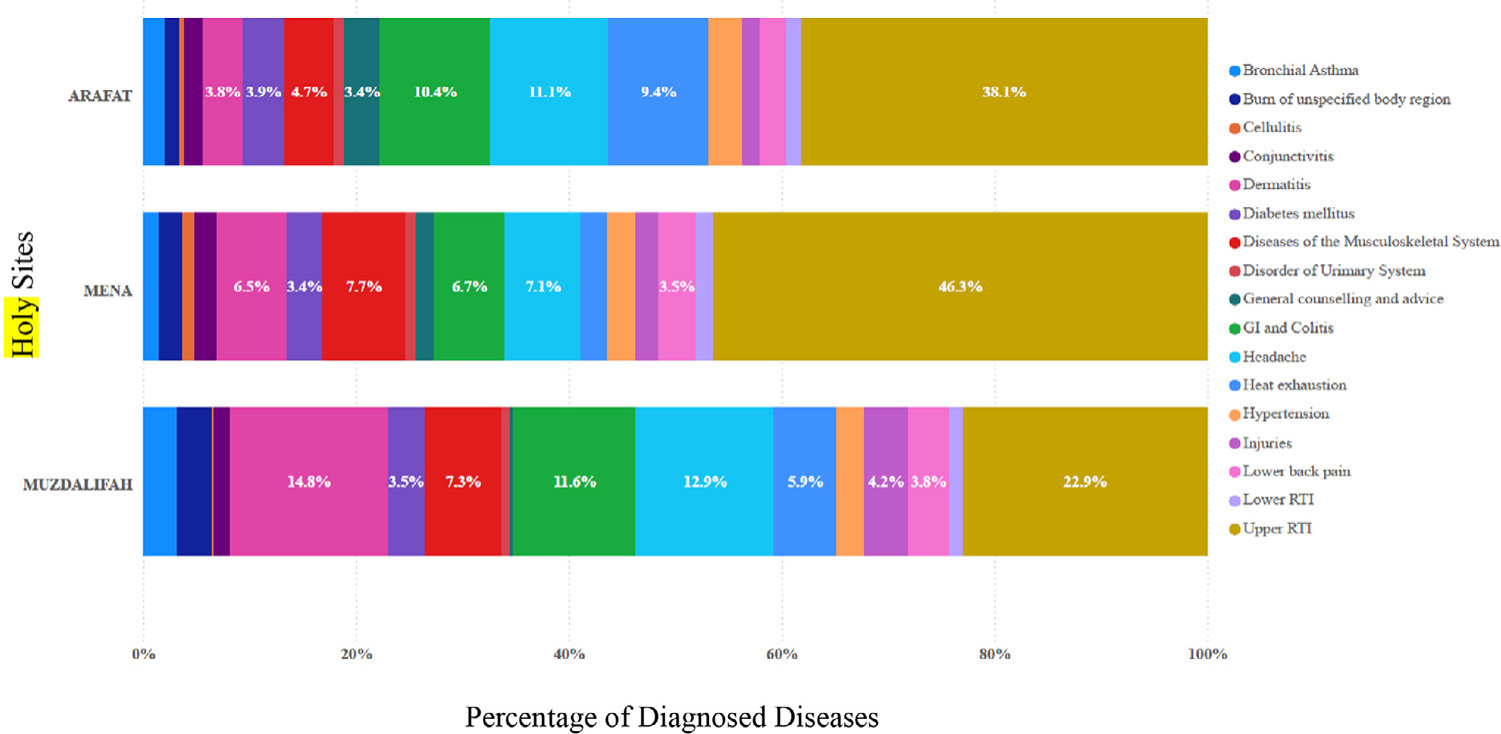
Percentage of diseases diagnosed at the outpatient clinics by holy sites during the 2024 Hajj.

The distribution of diseases also varied by location. In **Arafat**, URTIs were the most frequent diagnosis (38.1%), followed by headache (11.1%), GI disorders (10.4%), and heat exhaustion (9.4%). In **Mena**, URTI prevalence rose further to 46.3%, while musculoskeletal disorders (7.7%), headache (7.1%), and GI disorders (6.7%) were also common. In **Muzdalifah**, URTIs accounted for 22.9% of cases, but headache (12.9%) and GI disorders (11.6%) were proportionally higher than in other sites (Figure 3). These findings underscore the critical need for site‑specific and targeted healthcare strategies to effectively address the diverse health needs of pilgrims across different locations throughout their Hajj journey.

## 4. Discussion

Drawing on the analysis of over 37,000 patient records from the 2024 Hajj Health Services database, this study provides important insights into the demographic composition and evolving health demands of pilgrims. Older adults, especially those aged 60 and above, represented a substantial portion of healthcare users, reflecting a high burden of age‑related comorbidities and increased vulnerability to illness. Male pilgrims accounted for nearly two‑thirds of medical visits, while the participation of over 100 nationalities underscores the diversity and cultural complexity of care delivery. The high incidence of acute conditions—particularly URTIs in Mina—can be attributed to shared accommodations, crowd density, and environmental exposures. Additionally, the localized occurrence of heat exhaustion in Arafat and dermatological conditions in Muzdalifah highlights the need for flexible, site‑specific healthcare responses. These findings affirm the value of real‑time, data‑driven strategies to effectively meet the dynamic and context‑specific needs of pilgrims throughout the Hajj. These data‑driven findings are critical for informing targeted interventions and optimizing healthcare delivery during the Hajj pilgrimage.

### 4.1 Elderly pilgrims and chronic disease vulnerability

Older adults represent a particularly high‑risk group during the Hajj pilgrimage due to their increased burden of chronic illnesses. Studies indicate that nearly 87% of pilgrims over the age of 65 experience some form of morbidity, and 83% are classified as high health risk [10]. Common conditions such as stroke, diabetes, and coronary heart disease are especially prevalent among this group, making age a significant determinant of health vulnerability during Hajj.

Despite these risks, pre‑travel health screening and counseling services often fall short. This gap stems from poor coordination between healthcare providers and religious institutions, coupled with limited public health awareness campaigns. As a result, many pilgrims are inadequately prepared for the physical and environmental challenges of the pilgrimage. Notably, up to 20% of individuals experience delays or restrictions in their participation due to chronic illnesses such as kidney disease, dementia, and tuberculosis. These challenges are compounded by the lack of an integrated care system capable of managing complex, chronic conditions during mass gatherings [10].

### 4.2 Challenges in primary healthcare and NCD management

Non‑communicable diseases (NCDs) pose a growing challenge in Hajj health management, particularly within primary care settings in low‑ and middle‑income countries. Despite the increasing burden of chronic illnesses among pilgrims, many healthcare systems remain underprepared due to structural deficiencies. These include the absence of standardized clinical guidelines, insufficiently trained personnel, and limited healthcare resources.

To address these gaps, integrated and patient‑centered models of care—especially those leveraging digital health tools—have demonstrated potential in strengthening system resilience and enhancing health outcomes among vulnerable populations such as elderly pilgrims [11–13]. However, the success of such interventions depends on overcoming persistent barriers, including language and cultural differences, as well as infrastructural constraints, to ensure equitable and inclusive access to care.

### 4.3 Gender‑based barriers to healthcare access

Women face unique challenges in accessing healthcare during the Hajj pilgrimage, particularly due to insufficient provider communication and a lack of awareness regarding available services; this may explain why males accounted for the majority of medical visits in our study. In conservative contexts, these challenges are intensified by sociocultural norms, physical distance from health facilities, and a shortage of female healthcare professionals [14, 15]. Such barriers often result in delayed care, underreporting of symptoms, and reduced engagement with preventive services. Addressing these gender‑specific barriers requires a multifaceted approach, including the deployment of female medical staff, culturally responsive health communication, and outreach initiatives tailored to women’s needs in mass gatherings like Hajj.

### 4.4 Culturally competent care for a diverse pilgrim population

The multicultural nature of the Hajj, involving pilgrims from over 100 nationalities, demands a healthcare approach rooted in cultural competence. Effective care in this setting hinges on clear multilingual communication, access to qualified interpreters, and the provision of health education that is both culturally and linguistically appropriate. These components are vital not only for overcoming language barriers but also for fostering trust, improving patient engagement, and ensuring equitable access to healthcare. Studies have shown that culturally responsive interventions can significantly improve patient satisfaction, adherence to treatment, and health outcomes [16, 17]. In the complex environment of Hajj—marked by time constraints, crowd density, and cultural heterogeneity—such strategies are indispensable for safeguarding health and minimizing preventable complications.

### 4.5 Respiratory infections and infection control strategies

URTIs remain among the most prevalent health issues during Hajj, with reported incidence rates surpassing 44% in some cohorts [18–20]. The unique conditions of the pilgrimage—including extreme crowd density, communal accommodations, and prolonged physical proximity—create an ideal environment for the transmission of respiratory viruses such as rhinovirus and influenza [18, 20]. These factors not only elevate the risk of viral infections but also facilitate the spread of bacterial pathogens, posing a global public health concern. In response, a comprehensive infection control strategy is essential. This should include mandatory pre‑travel influenza vaccination, widespread promotion of face mask use, enhanced hygiene awareness campaigns, and on‑site surveillance system [21, 22]. Timely implementation of these measures can significantly reduce infection rates and help protect both local and international pilgrims.

### 4.6 Geographically driven health risks across Hajj sites

The Hajj pilgrimage exposes participants to a diverse range of health risks that differ significantly across key locations, influenced by environmental stressors, infrastructure limitations, and the dynamics of large‑scale human congregation. These variations highlight the necessity of geographically adaptive healthcare strategies tailored to each site’s unique conditions.

**Arafat** is particularly associated with heat‑related illnesses—especially heat exhaustion—resulting from extreme temperatures, direct sun exposure, and physically strenuous rituals. Elderly individuals, those with chronic conditions, and first‑time pilgrims from temperate regions face the greatest vulnerability [23–25].

**Muzdalifah**, in contrast, sees a higher incidence of skin‑related issues such as dermatitis and cellulitis, likely due to prolonged outdoor exposure, contact with dust, and restricted access to hygiene facilities [23].

**Mena** is characterized by widespread URTIs consistent with our findings, attributed to the dense, shared living arrangements that promote the rapid transmission of infectious agents. More than half of pilgrims are reported to develop respiratory symptoms post‑rituals in this location [18, 23, 26].

These spatial variations in disease burden underscore the critical need for location‑specific health interventions. Moreover, the pattern of primary healthcare utilization closely follows the movement and congregation of pilgrims, with demand peaking at high‑density sites such as Mena [7, 27]. In this dynamic context, healthcare delivery models must be flexible, scalable, and responsive to real‑time conditions to effectively mitigate health risks and ensure service continuity.

### 4.7 Preventive strategies and health promotion

Health conditions commonly encountered during the Hajj are largely preventable or manageable through well‑structured public health preparedness and proactive preventive strategies. Central to these efforts is vaccination, which remains a cornerstone of disease prevention. Updated immunization protocols and rigorous pre‑travel vaccination campaigns—especially for meningococcal disease, influenza, and COVID‑19—are vital in mitigating the transmission of communicable diseases [4, 28–29].

Despite these measures, many pilgrims remain unaware of potential health risks and the necessary preventive actions. This highlights the importance of pre‑travel counseling that reinforces key practices such as adequate hydration, personal hygiene, medication adherence, and early recognition of symptoms. Such education empowers individuals to engage in self‑care and reduces pressure on healthcare services [29–31].

Equally important is the role of health communication both before and during the pilgrimage. Promoting protective behaviors—such as hand hygiene, mask usage, and social distancing—has been shown to significantly lower the incidence of respiratory and other infectious diseases [31–33].

Furthermore, robust outbreak preparedness necessitates the implementation of real‑time surveillance systems and rapid response mechanisms. The loss of over 28,000 health records due to incomplete data emphasizes the urgent need for standardized, high‑quality electronic data collection systems. The integration of advanced analytics can further support timely decision‑making and effective public health interventions during mass gatherings [4, 34–35].

### 4.8 Strengths and limitations

There are numerous noteworthy strengths to this study. Using a sizable dataset of 37,758 patient records from the Saudi Ministry of Health’s Hajj Health Services database provided thorough insights into healthcare utilization during the 2024 Hajj as well as strong statistical power. The inclusion of pilgrims from more than 100 different nationalities reflects the pilgrimage’s multicultural nature and improves the findings’ generalizability. Furthermore, the analysis of condition patterns that are specific to both time and place offers important proof in favor of focused public health initiatives and adaptive resource allocation throughout Mena, Arafat, and Muzdalifah.

Nonetheless, it is important to recognize some limitations. In addition to excluding causal inference, the retrospective cross‑sectional approach fails to record longitudinal or post‑Hajj health consequences. Incomplete clinical or demographic information also led to the exclusion of more than 28,000 records, which may have introduced selection bias. The capacity to evaluate risk stratification among vulnerable groups is hampered by the absence of comprehensive clinical data on comorbidities, medication use, and illness severity. Lastly, the scope of interpretation is further limited by the lack of contextual factors such as health practices, immunization status, and environmental circumstances, as well as possible underreporting by pilgrims who did not use Ministry of Health’s facilities.

## 5. Conclusion

The findings from the 2024 Hajj season underscore the complex and multifaceted nature of healthcare delivery during mass gatherings. Pilgrims face a broad spectrum of health risks—ranging from communicable and chronic diseases to gender‑based and cultural barriers—shaped by demographic, environmental, and logistical factors. Older adults, mainly those aged 60 and above, exemplified a substantial portion of healthcare users, highlighting a high burden of age‑related illness and increased vulnerability to diseases. Furthermore, male pilgrims accounted for nearly two‑thirds of medical visits, while participation from over 100 nationalities represents the diversity and complexity of care delivery. The high burden of preventable conditions highlights persistent gaps in pre‑travel preparation, multilingual support, and site‑specific medical services. According to this study, different holy sites present different dominant health risks (heat in Arafat, URTIs in Mina), requiring site‑related resources. Respiratory infections remain the major burden of diseases; thus, comprehensive prevention like vaccination, masks, hygiene is necessary to manage transmission. To ensure equitable and effective healthcare, Hajj planning must prioritize integrated public health strategies that include robust surveillance, context‑sensitive interventions, and scalable infrastructure. Addressing data quality, enhancing real‑time responsiveness, and tailoring services to the unique dynamics of each sacred site will be essential for safeguarding the health of pilgrims in future seasons.

## Data Availability

Data were obtained from the Population Health Management Department of the Makkah Health Cluster. These data are not publicly available and were provided to the researchers upon request.

## Additional File

The additional file for this article can be found as follows:

Supplementary Table 1. Characteristics of patients attending the outpatient clinics by the most common diagnoses during the 2024 Hajj.
