# Supplementary material for: Disease Burden and Pattern of Healthcare Utilization Among Pilgrims During Hajj 2024: A Cross‑Sectional Analysis
Source: Ann Glob Health. 2026 Mar 9;92(1):25. doi: 10.5334/aogh.4956 (PMC12985899; doi:10.5334/aogh.4956)
Supplement: Supplementary Table 1. — Characteristics of patients attending the outpatient clinics by the most common diagnoses during the 2024 Hajj. [file agh-92-1-4956-s1.pdf]

Supplementary Table 1: Characteristics of patients attending the outpatient clinics by the most common diagnoses during the 2024 Hajj

	Diseases (%)																	
	**N**	**URTI**	**Head-ache**	**GI and Colitis**	**Musculo-skeletal**	**Derma**	**HE**	**DM**	**LBP**	**HTN**	**Burn**	**Injuries**	**Conj**	**Counselling**	**LRTI**	**BA**	**Cellulitis**	**DUS**
**Gender**																		
Male	24,744	45.98	7.22	6.07	6.88	7.63	2.74	3.52	3.33	2.44	2.38	2.59	2.09	1.88	1.70	1.44	1.19	0.90
Female	13,014	41.85	8.94	9.78	8.04	3.75	5.33	3.42	3.47	3.33	1.66	1.12	1.91	2.17	1.60	1.92	0.69	1.03
Chi2	--	59.05	35.11	172.98	16.98	218.14	162.86	0.26	0.45	25.08	21.44	90.14	1.51	3.83	0.55	12.56	21.18	1.50
P-value	--	**<0.001**	**<0.001**	**<0.001**	**<0.001**	**<0.001**	**<0.001**	0.612	0.502	**<0.001**	**<0.001**	**<0.001**	0.219	**0.050**	0.457	**<0.001**	**<0.001**	0.220
**Age (Years)**																		
16-25	1,362	46.55	10.28	9.47	4.55	7.20	3.82	0.44	1.40	0.07	2.06	2.72	1.98	3.30	1.25	2.57	1.10	1.25
26-30	1,711	47.05	8.65	9.82	6.08	8.94	2.05	0.64	2.51	0.41	2.22	1.93	1.34	2.86	1.46	1.93	0.99	1.11
31-35	2,879	43.94	8.93	8.34	7.43	9.90	2.43	1.08	3.37	1.01	2.29	2.19	1.56	2.15	1.29	1.91	1.15	1.04
36-40	3,864	46.38	8.10	7.19	7.04	8.80	3.16	1.48	3.23	1.06	3.08	2.04	1.45	1.86	1.53	1.53	1.04	1.04
41-45	4,358	46.37	8.38	6.33	6.56	7.99	2.34	2.68	3.19	2.39	2.27	2.02	1.90	1.74	2.09	1.54	1.01	1.19
46-50	4,383	45.86	8.21	5.95	7.07	6.80	2.90	3.26	3.56	2.81	2.56	1.94	2.08	1.60	1.69	1.37	1.14	1.21
51-55	4,577	45.47	8.06	6.71	7.17	6.62	3.60	3.74	4.02	2.67	2.18	1.84	1.94	1.42	1.55	1.38	1.11	0.52
56-60	4,653	45.65	6.86	6.90	8.17	4.36	4.10	4.30	3.37	3.83	2.04	1.68	2.13	1.76	1.74	1.31	0.90	0.90
> 60	9,971	41.04	6.82	7.96	7.94	3.50	5.08	5.82	3.57	4.33	1.49	2.41	2.54	2.28	1.75	1.74	0.93	0.80
Chi2	--	74.68	41.41	57.40	34.72	311.97	117.44	354.78	28.39	261.61	40.89	13.87	27.48	37.50	10.56	17.81	2.99	19.50
P-value	--	**<0.001**	**<0.001**	**<0.001**	**<0.001**	**<0.001**	**<0.001**	**<0.001**	**<0.001**	**<0.001**	**<0.001**	0.085	**0.001**	**<0.001**	0.228	**0.023**	0.935	**0.012**
**Nationality**																		
Afghanistan	832	32.45	18.63	11.18	10.46	4.09	3.13	1.44	6.01	2.52	0.96	1.68	0.48	0.96	1.44	1.56	1.32	1.68
Algeria	1,459	30.71	6.65	5.21	7.61	10.90	4.25	4.52	1.78	3.50	4.59	6.44	1.51	3.70	1.17	1.10	6.03	0.34
Bangladesh	1,071	30.35	7.38	8.40	9.71	9.43	2.89	7.56	5.42	5.23	1.12	2.43	3.17	1.40	2.61	1.12	0.56	1.21
Egypt	5,180	41.04	7.51	7.45	6.85	5.89	6.49	4.98	2.64	3.42	2.90	1.64	1.68	2.49	1.37	1.91	0.98	0.75
India	3,958	37.77	6.24	10.76	10.74	7.28	5.94	2.88	2.80	2.17	2.73	2.43	1.97	1.74	1.64	1.29	0.56	1.06
Iran	3,891	63.20	5.06	3.88	6.58	4.03	1.95	1.36	4.50	1.64	0.87	0.31	1.90	1.05	2.08	0.67	0.18	0.72
Iraq	788	35.41	6.98	5.58	8.50	6.60	4.70	6.98	4.19	7.11	2.92	2.66	1.40	1.65	1.90	1.14	1.02	1.27
Jordan	851	56.17	5.99	5.41	4.00	4.00	5.29	3.41	2.59	2.82	2.59	1.06	1.65	0.47	1.76	1.29	0.35	1.18
Libya	812	42.98	7.39	5.42	5.30	4.31	4.80	7.88	2.71	5.42	1.72	1.85	1.97	1.72	1.97	0.99	2.71	0.86
Morocco	2,230	47.94	7.40	6.59	5.56	6.19	2.51	3.54	2.65	2.11	1.84	3.77	2.29	2.20	1.61	1.30	1.35	1.17
Niger	584	51.20	6.16	10.27	14.90	2.91	0.34	1.54	1.71	0.34	0.17	0.68	4.11	0.17	2.74	1.37	0.17	1.20
Nigeria	2,756	57.84	13.75	6.93	2.90	2.14	0.33	0.51	7.51	1.63	0.36	0.58	2.54	0.22	1.20	0.80	0.22	0.54
Other	4,100	45.63	8.76	7.44	5.34	6.10	4.29	3.22	2.93	2.56	2.20	1.73	2.49	1.73	1.76	1.95	1.00	0.88
Pakistan	4,203	41.90	5.95	7.28	9.28	10.35	3.19	2.90	2.93	1.95	2.31	2.78	1.83	2.12	2.00	1.14	0.86	1.24
Saudi Arabia	3,559	40.32	8.82	7.11	7.39	6.18	2.16	3.93	2.16	3.54	2.61	2.36	2.02	4.19	1.29	3.82	1.12	0.96
Sudan	494	47.77	6.28	6.07	5.67	3.85	1.82	8.50	2.63	3.85	0.40	2.02	2.83	2.02	1.62	2.83	0.40	1.42
Turkey	379	40.11	13.46	20.05	1.32	1.06	2.37	2.11	2.64	2.64	1.06	3.17	1.58	1.58	1.85	2.37	2.11	0.53
Yamen	611	29.13	5.89	8.18	11.46	11.46	1.96	6.22	3.76	3.60	4.91	2.78	1.64	3.27	1.15	2.45	0.49	1.64
Chi2	--	1.3e+03	387.65	280.84	340.17	391.66	381.07	373.21	250.24	188.66	196.81	294.99	49.61	215.53	31.03	176.10	462.12	32.78
***P*-value**	--	**<0.001**	**<0.001**	**<0.001**	**<0.001**	**<0.001**	**<0.001**	**<0.001**	**<0.001**	**<0.001**	**<0.001**	**<0.001**	**<0.001**	**<0.001**	**0.020**	**<0.001**	**<0.001**	**<0.001**

URTI=Upper Respiratory Tract Infection; **GI and Colitis**=Gastrointestinal and Colitis; **Musculo-skeletal**=Diseases of the Musculoskeletal System; **Derma**=Dermatitis; **HE**=Heat exhaustion; **DM**=Diabetes Mellitus; **LBP**=Lower Back Pain; **HTN**=Hypertension; **Burn**=Burn of unspecified body region; **Conj**=Conjunctivitis; C**ounselling**=General counselling and advice; **LRTI**=Lower Respiratory Tract Infection; **BA**=Bronchial Asthma; **DUS**=Disorder of Urinary System
